# Supplementary figures and images for: Dalpiciclib partially abrogates ER signaling activation induced by pyrotinib in HER2+HR+ breast cancer
Source: eLife. 2023 Jan 5;12:e85246. doi: 10.7554/eLife.85246 (PMC9822241; doi:10.7554/eLife.85246)

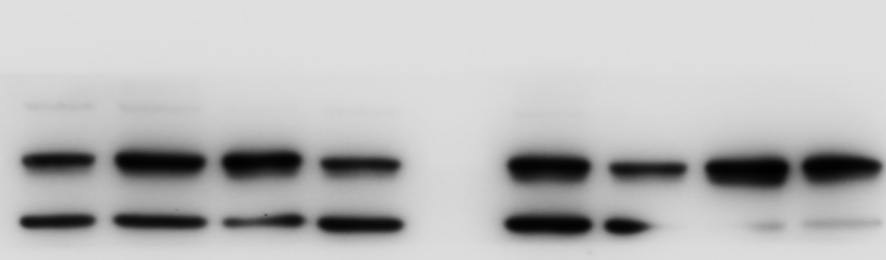

Supplement: Figure 2—figure supplement 1—source data 1. [file elife-85246-fig2-figsupp1-data1.zip › Figure 2-sigure supplement 1 source data 1/ER(nuclear).tif]

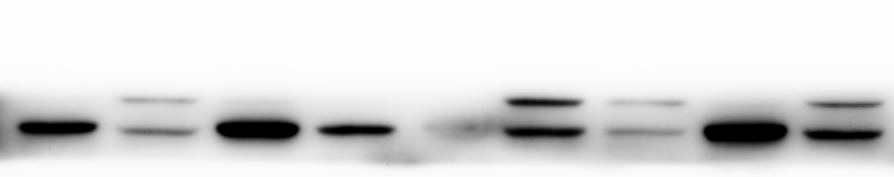

Supplement: Figure 2—figure supplement 1—source data 1. [file elife-85246-fig2-figsupp1-data1.zip › Figure 2-sigure supplement 1 source data 1/ERú¿total).tif]

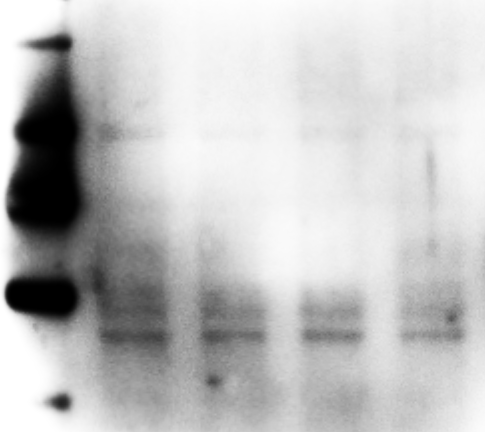

Supplement: Figure 2—figure supplement 1—source data 1. [file elife-85246-fig2-figsupp1-data1.zip › Figure 2-sigure supplement 1 source data 1/ER.tif]

Raw gels of Figure 2-figure supplement 1 a, b and d

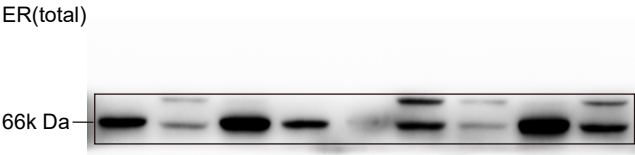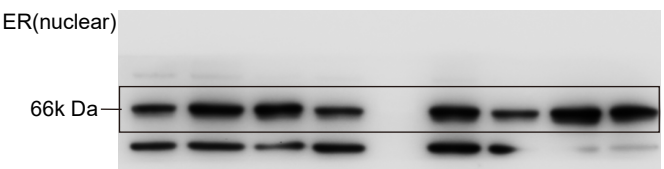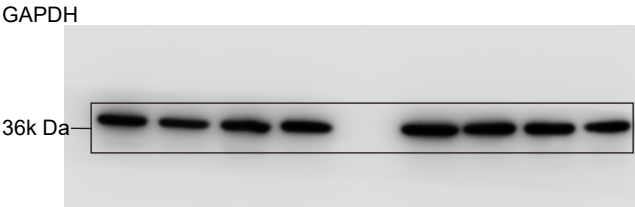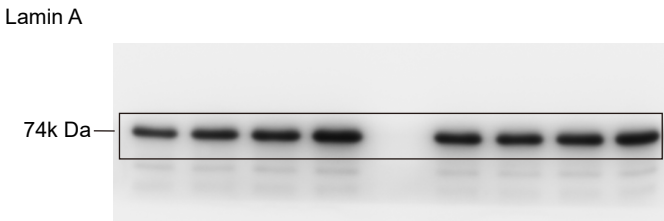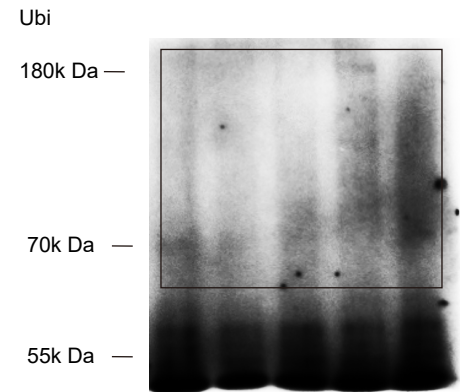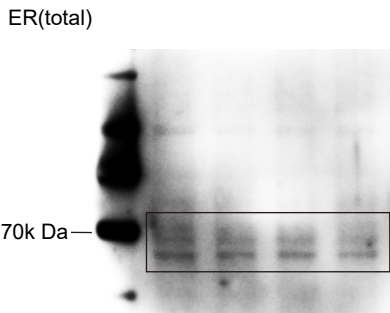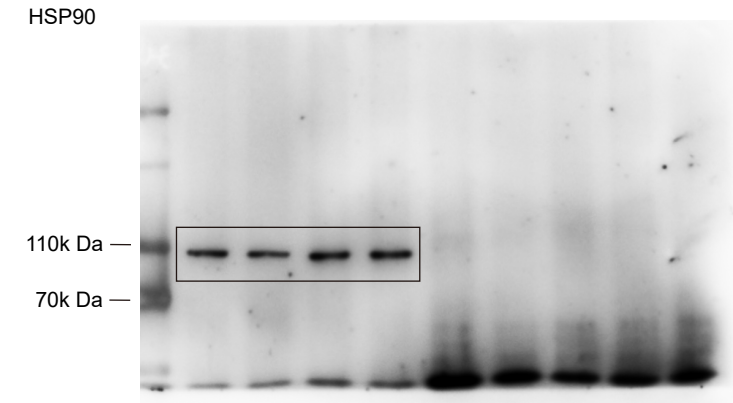

Supplement: Figure 2—figure supplement 1—source data 1. [file elife-85246-fig2-figsupp1-data1.zip › Figure 2-sigure supplement 1 source data 1/Figure 2-figure supplement 1 a b and d.pdf]

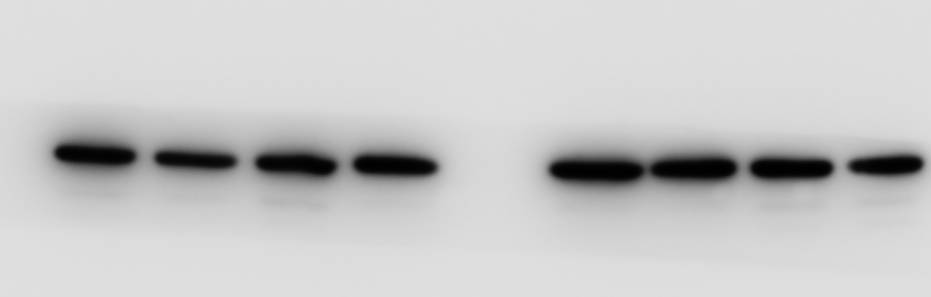

Supplement: Figure 2—figure supplement 1—source data 1. [file elife-85246-fig2-figsupp1-data1.zip › Figure 2-sigure supplement 1 source data 1/GAPDH.tif]

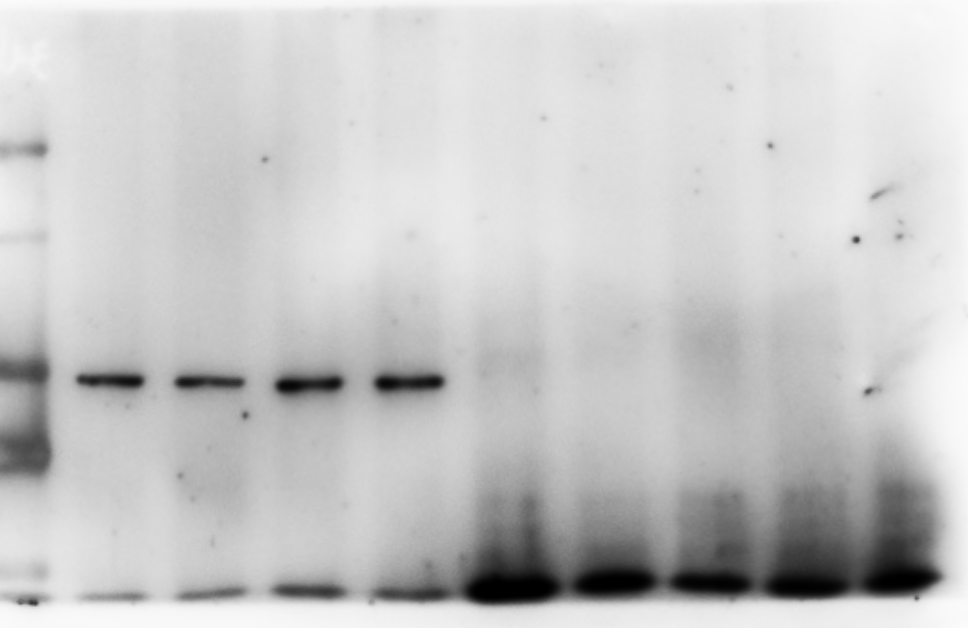

Supplement: Figure 2—figure supplement 1—source data 1. [file elife-85246-fig2-figsupp1-data1.zip › Figure 2-sigure supplement 1 source data 1/HSP90.tif]

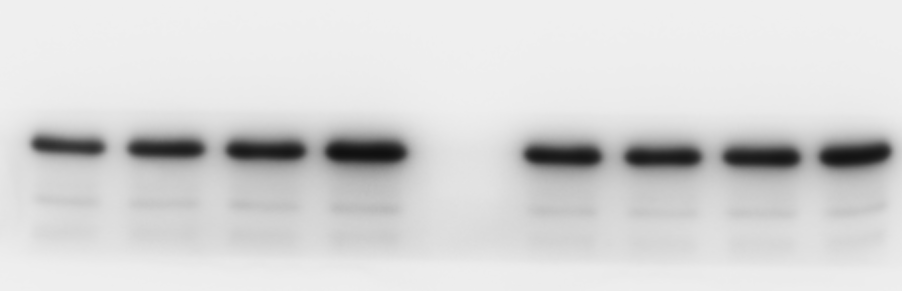

Supplement: Figure 2—figure supplement 1—source data 1. [file elife-85246-fig2-figsupp1-data1.zip › Figure 2-sigure supplement 1 source data 1/Lamin A .tif]

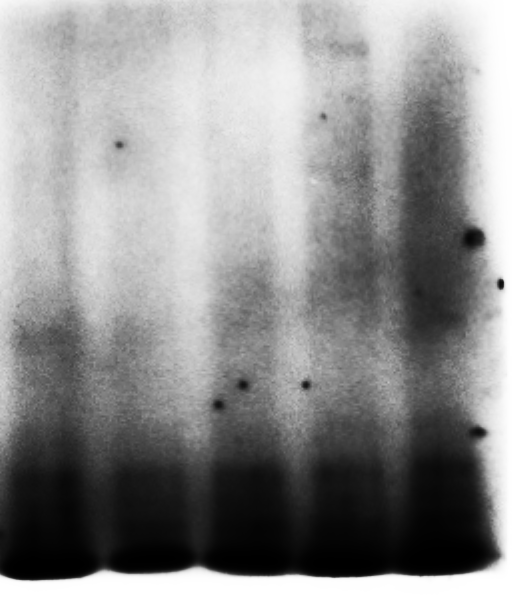

Supplement: Figure 2—figure supplement 1—source data 1. [file elife-85246-fig2-figsupp1-data1.zip › Figure 2-sigure supplement 1 source data 1/Ubi .tif]

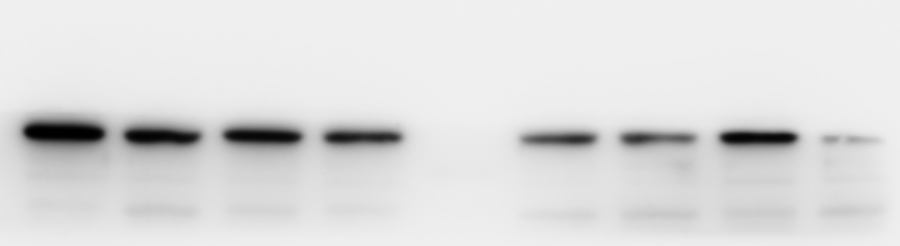

Supplement: Figure 4—source data 1. [file elife-85246-fig4-data1.zip › Figure 4 source data 1/AKTtif.tif]

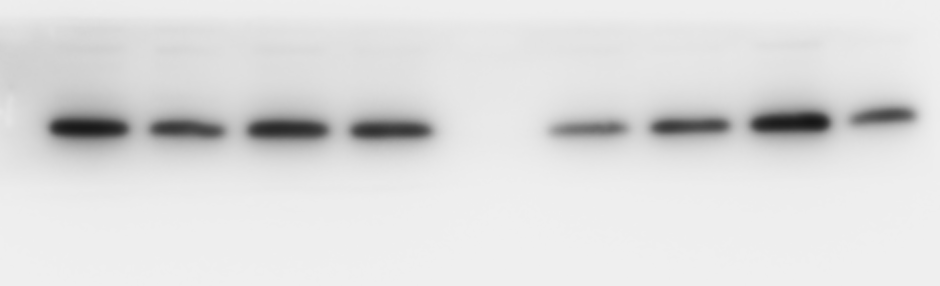

Supplement: Figure 4—source data 1. [file elife-85246-fig4-data1.zip › Figure 4 source data 1/CDK4.tif]

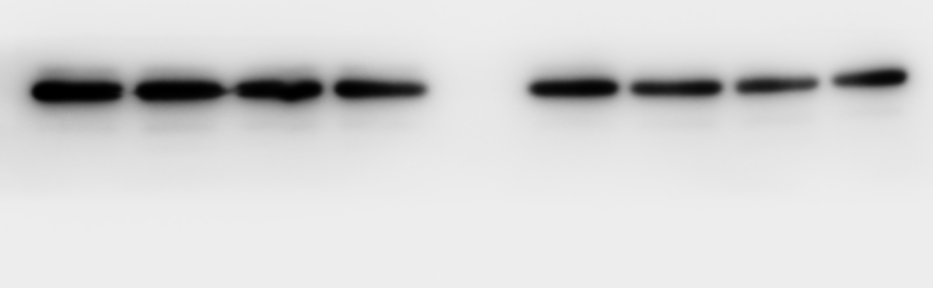

Supplement: Figure 4—source data 1. [file elife-85246-fig4-data1.zip › Figure 4 source data 1/CDK6.tif]

## Raw gels of Figure 4 a

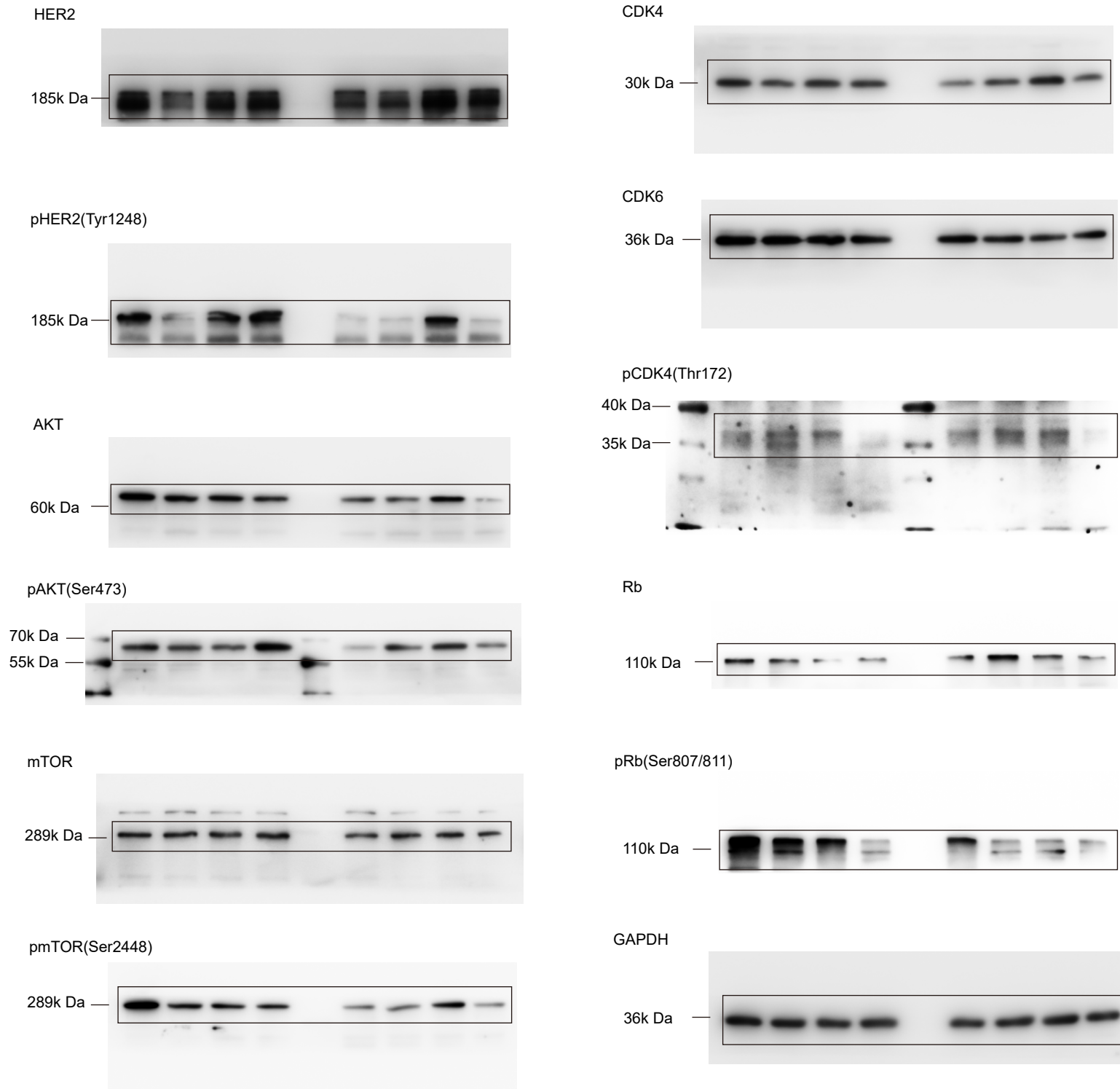

Supplement: Figure 4—source data 1. [file elife-85246-fig4-data1.zip › Figure 4 source data 1/Figure 4 a.pdf]

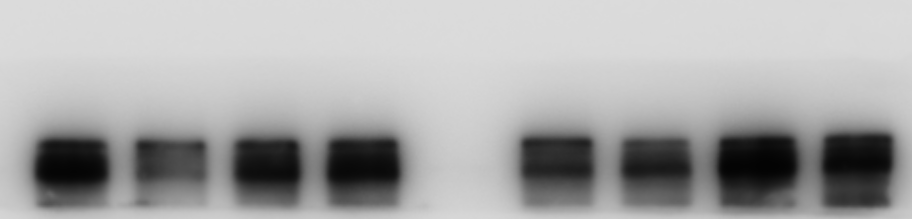

Supplement: Figure 4—source data 1. [file elife-85246-fig4-data1.zip › Figure 4 source data 1/HER2 .tif]

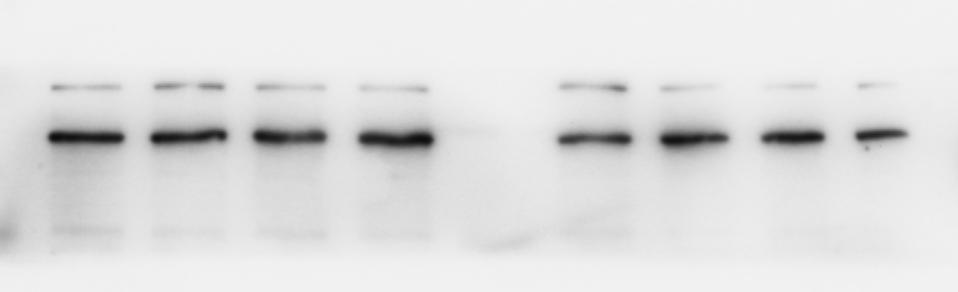

Supplement: Figure 4—source data 1. [file elife-85246-fig4-data1.zip › Figure 4 source data 1/mTOR.tif]

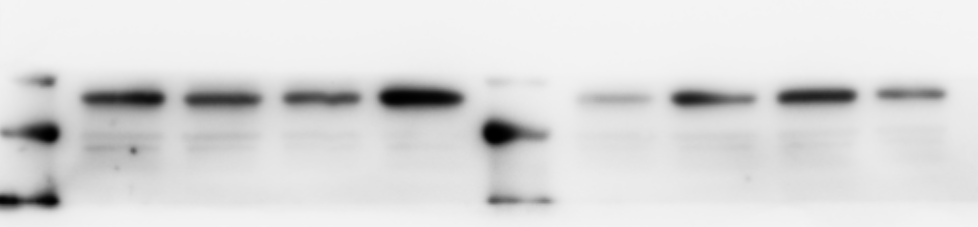

Supplement: Figure 4—source data 1. [file elife-85246-fig4-data1.zip › Figure 4 source data 1/pAKT.tif]

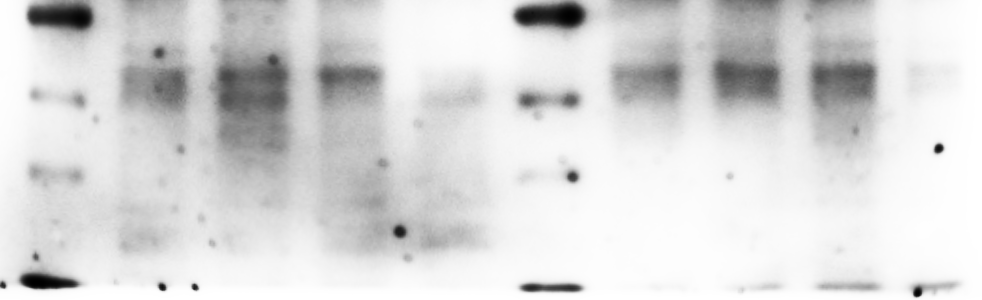

Supplement: Figure 4—source data 1. [file elife-85246-fig4-data1.zip › Figure 4 source data 1/pCDK4.tif]

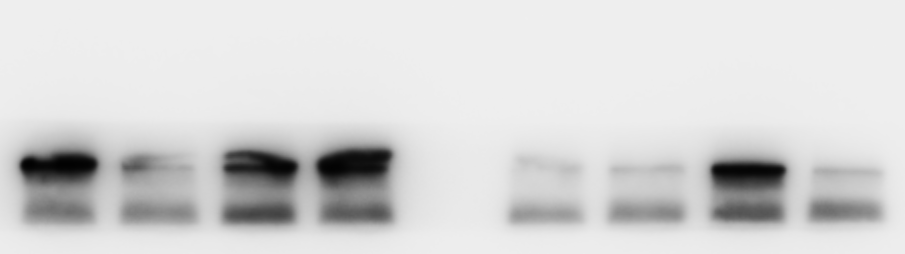

Supplement: Figure 4—source data 1. [file elife-85246-fig4-data1.zip › Figure 4 source data 1/pHER2.tif]

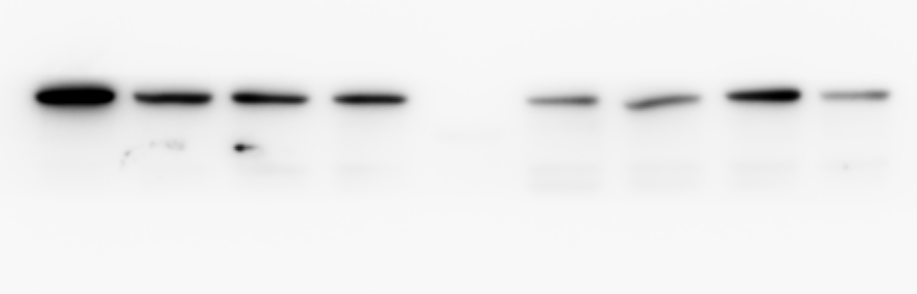

Supplement: Figure 4—source data 1. [file elife-85246-fig4-data1.zip › Figure 4 source data 1/pmTOR.tif]

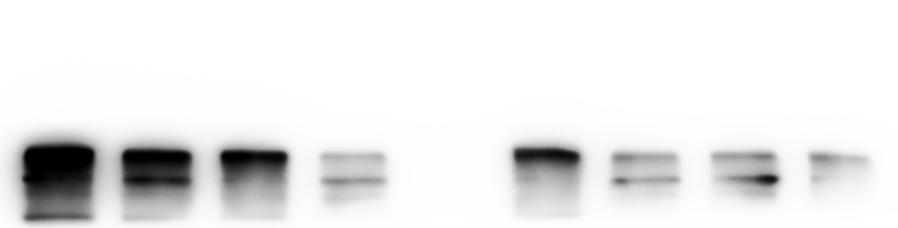

Supplement: Figure 4—source data 1. [file elife-85246-fig4-data1.zip › Figure 4 source data 1/pRb.tif]

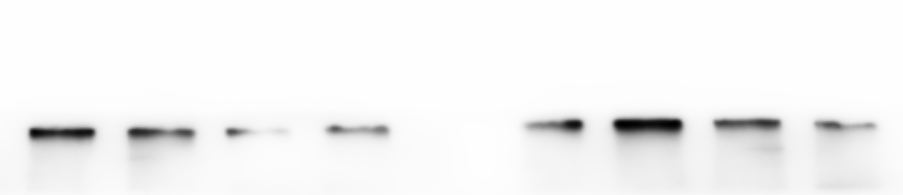

Supplement: Figure 4—source data 1. [file elife-85246-fig4-data1.zip › Figure 4 source data 1/Rb.tif]
