# Supplementary material for: Dalpiciclib partially abrogates ER signaling activation induced by pyrotinib in HER2+HR+ breast cancer
Source: eLife. 2023 Jan 5;12:e85246. doi: 10.7554/eLife.85246 (PMC9822241; doi:10.7554/eLife.85246)

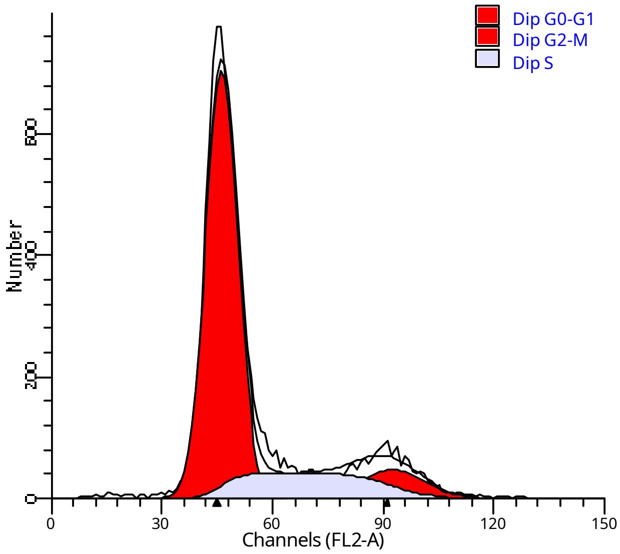

Date: 04-Jan-70 File: Data.001
Model:
%G0-G1: 72.14 at 46.18
%S: 18.19
%G2-M: 9.67 at 92.36
%CV: 9.37
RCS: 3.612

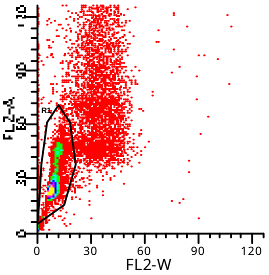

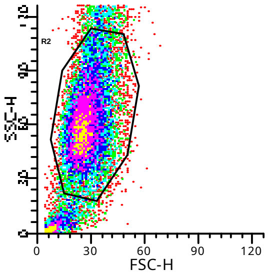

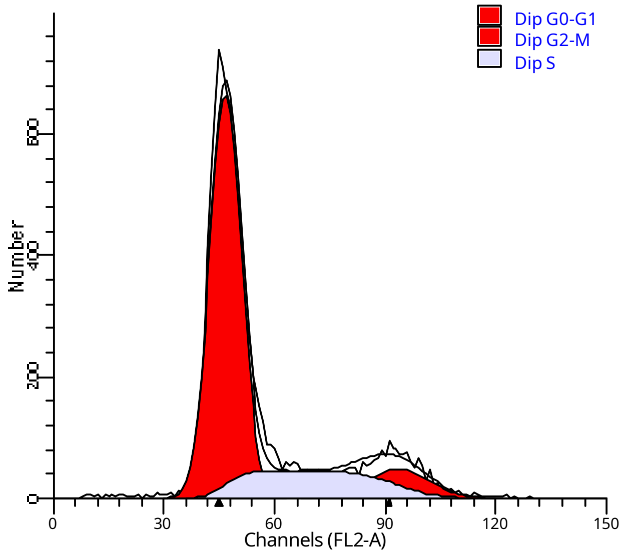

Date: 04-Jan-70 File: Data.002
Model:
%G0-G1: 69.55 at 46.68
%S: 20.50
%G2-M: 9.95 at 93.36
%CV: 9.16
RCS: 2.993

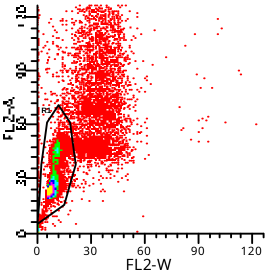

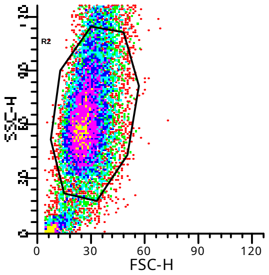

ModFitLT V3.0(PMac)

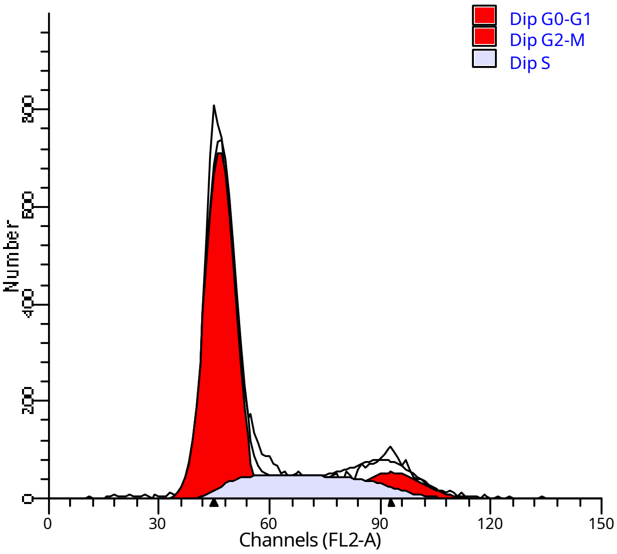

Date: 04-Jan-70 File: Data.003
Model:
%G0-G1: 69.11 at 46.50
%S: 20.61
%G2-M: 10.29 at 93.01
%CV: 8.54
RCS: 3.209

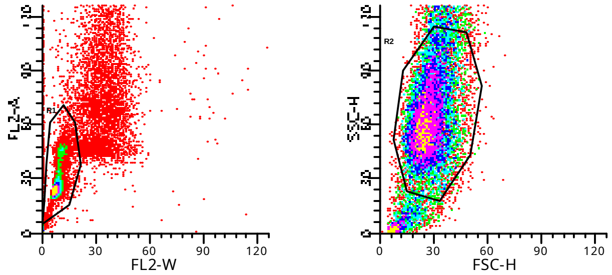

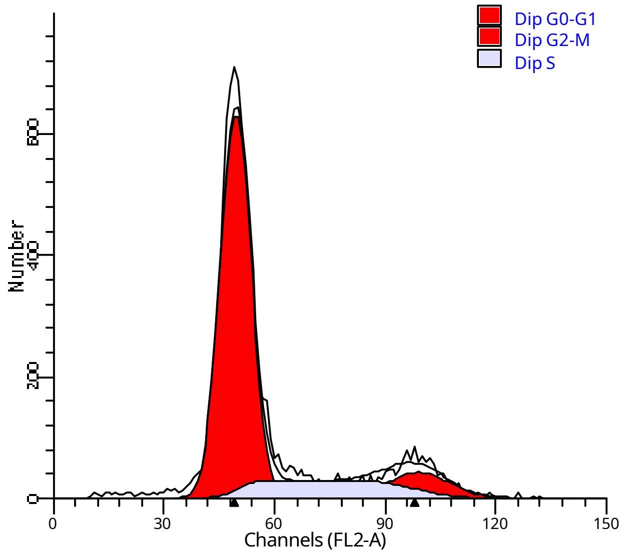

Date: 04-Jan-70 File: Data.004
Model:
%G0-G1: 73.86 at 49.46
%S: 16.18
%G2-M: 9.96 at 98.92
%CV: 8.43
RCS: 4.114

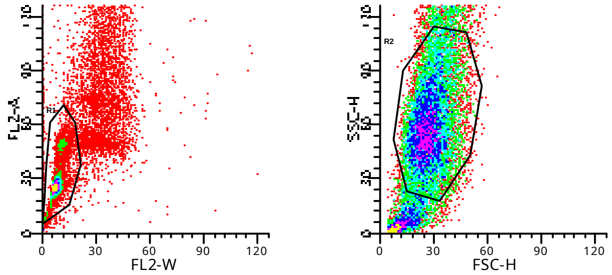

ModFitLT V3.0(PMac)

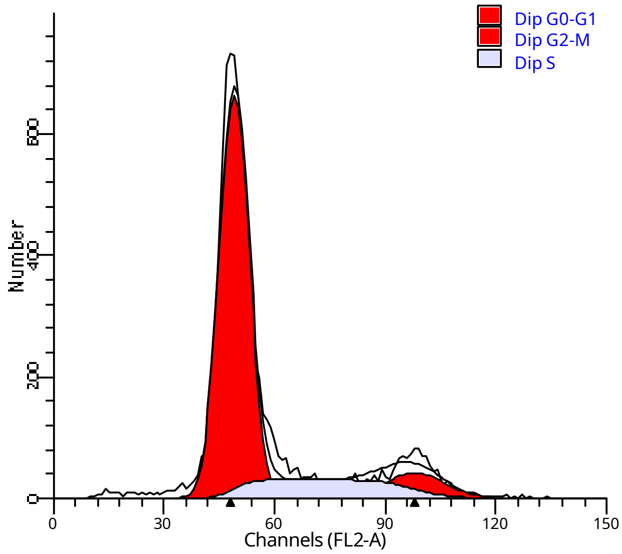

Date: 04-Jan-70 File: Data.005
Model:
%G0-G1: 73.86 at 49.04
%S: 16.76
%G2-M: 9.38 at 98.08
%CV: 8.25
RCS: 4.641

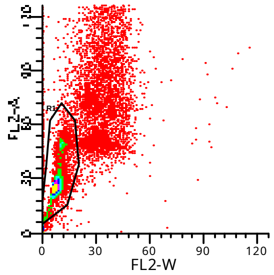

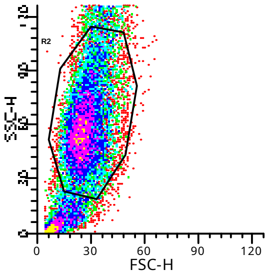

ModFitLT V3.0(PMac)

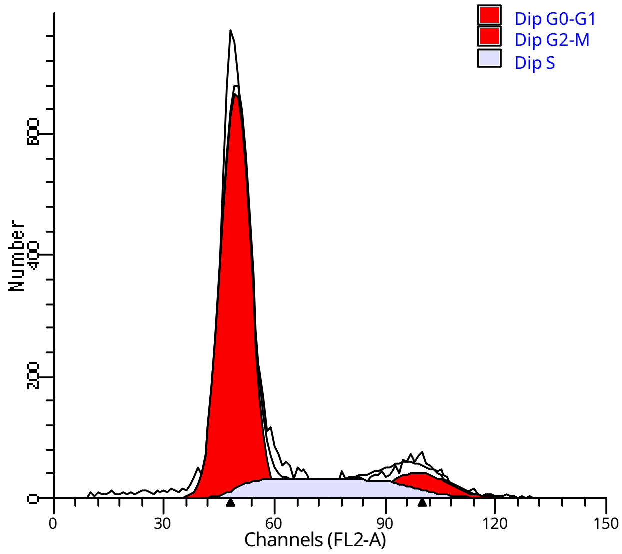

Date: 04-Jan-70 File: Data.006
Model:
%G0-G1: 73.58 at 49.41
%S: 17.31
%G2-M: 9.11 at 98.81
%CV: 8.02
RCS: 4.462

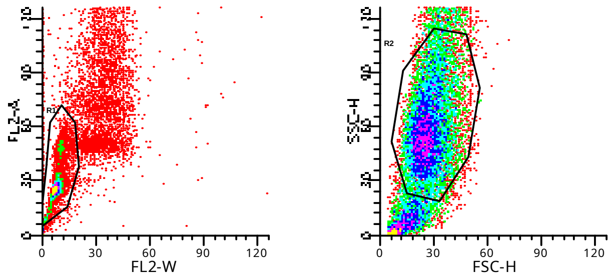

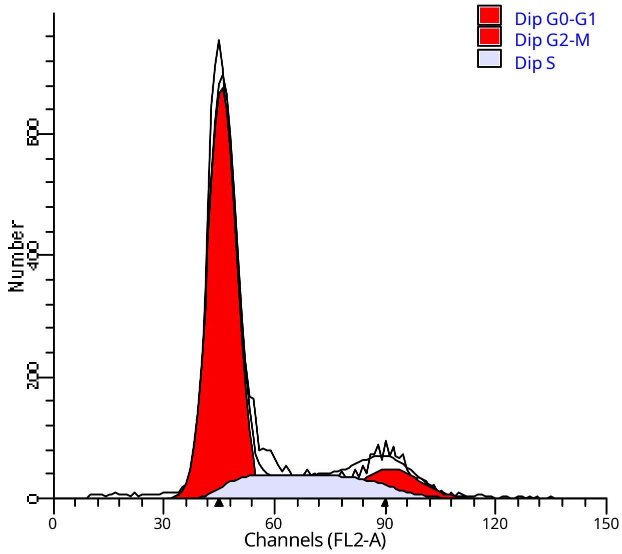

Date: 04-Jan-70 File: Data.007
Model:
%G0-G1: 70.94 at 45.75
%S: 18.97
%G2-M: 10.09 at 91.49
%CV: 8.31
RCS: 4.300

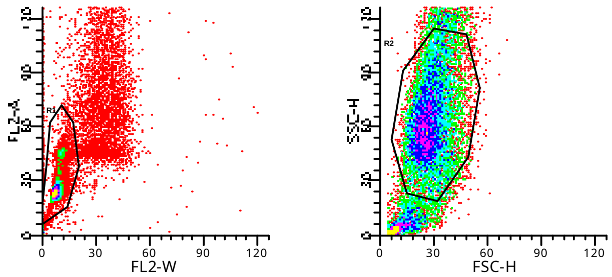

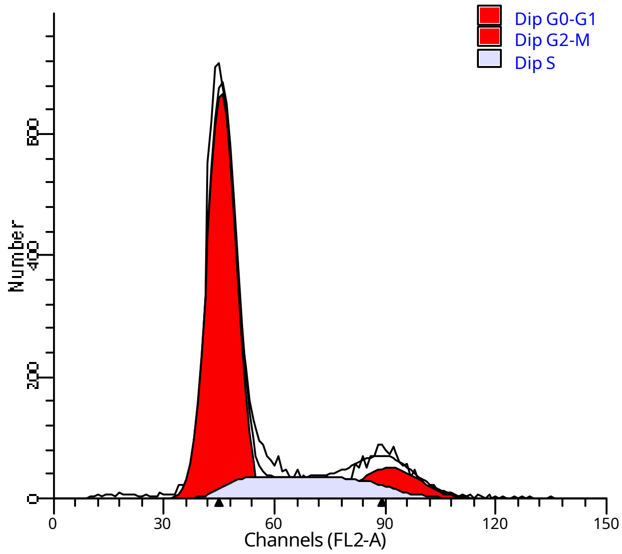

Date: 04-Jan-70 File: Data.008
Model:
%G0-G1: 71.37 at 45.62
%S: 17.74
%G2-M: 10.89 at 91.23
%CV: 8.47
RCS: 3.610

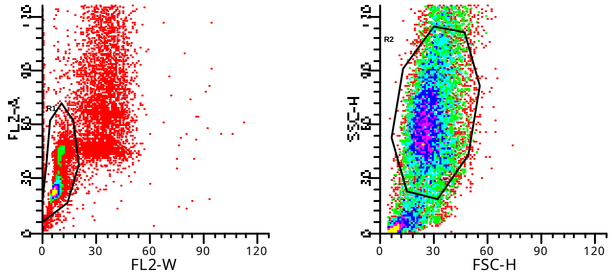

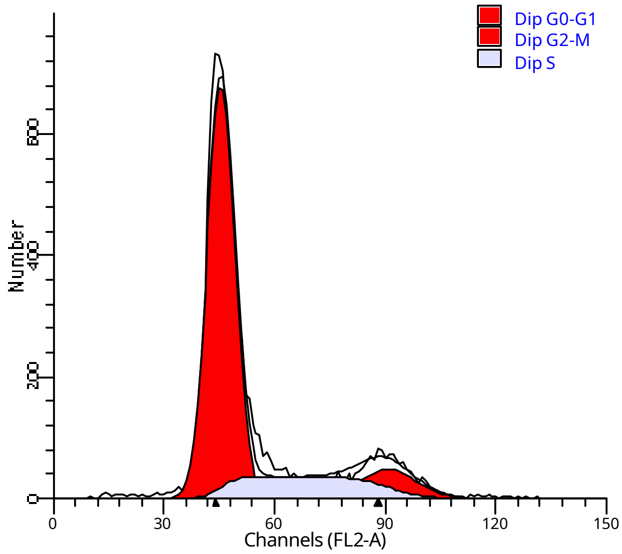

Date: 04-Jan-70 File: Data.009
Model:
%G0-G1: 71.61 at 45.44
%S: 18.41
%G2-M: 9.98 at 90.89
%CV: 8.21
RCS: 3.347

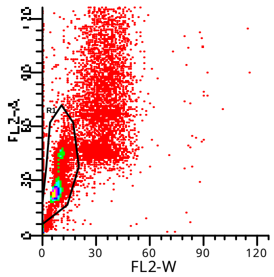

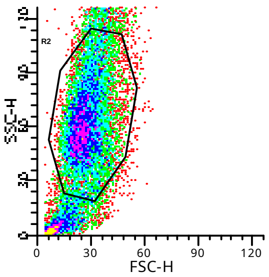

ModFitLT V3.0(PMac)

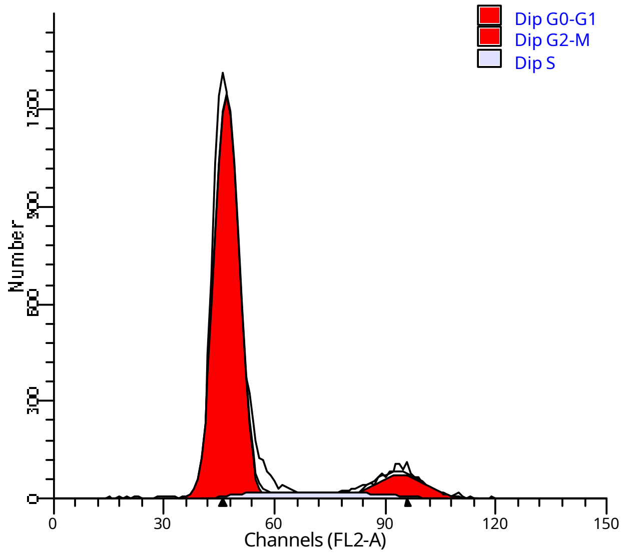

Date: 04-Jan-70 File: Data.010
Model:
%G0-G1: 83.47 at 46.99
%S: 6.59
%G2-M: 9.94 at 93.98
%CV: 6.92
RCS: 6.051

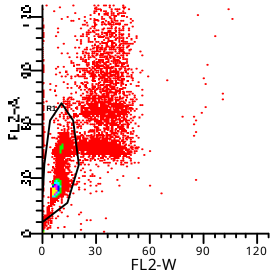

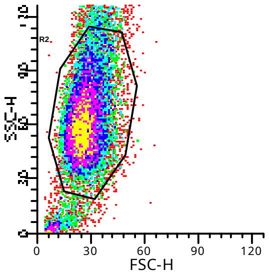

ModFitLT V3.0(PMac)

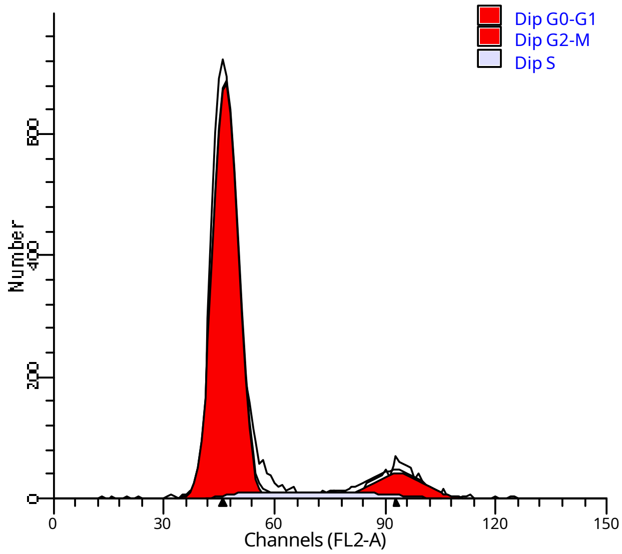

Date: 04-Jan-70 File: Data.011
Model:
%G0-G1: 82.98 at 46.68
%S: 7.08
%G2-M: 9.94 at 93.36
%CV: 7.16
RCS: 3.114

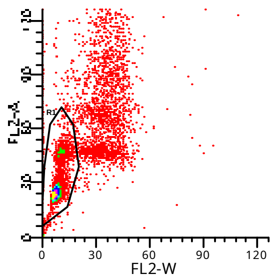

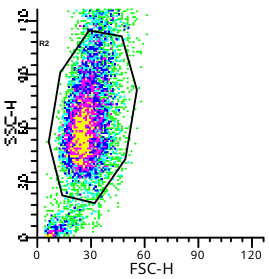

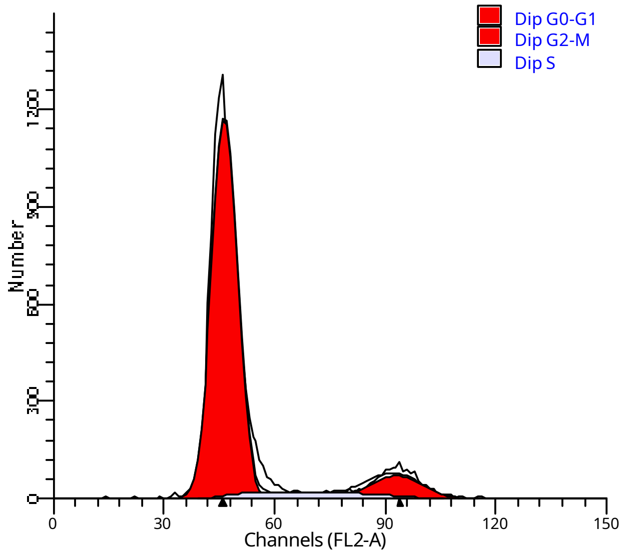

Date: 04-Jan-70 File: Data.012
Model:
%G0-G1: 83.42 at 46.39
%S: 6.54
%G2-M: 10.05 at 92.78
%CV: 7.42
RCS: 5.437

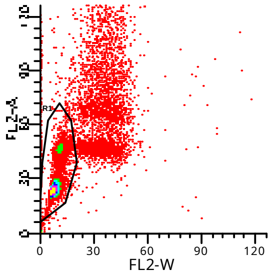

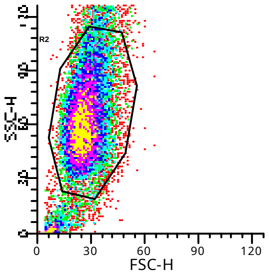

ModFitLT V3.0(PMac)

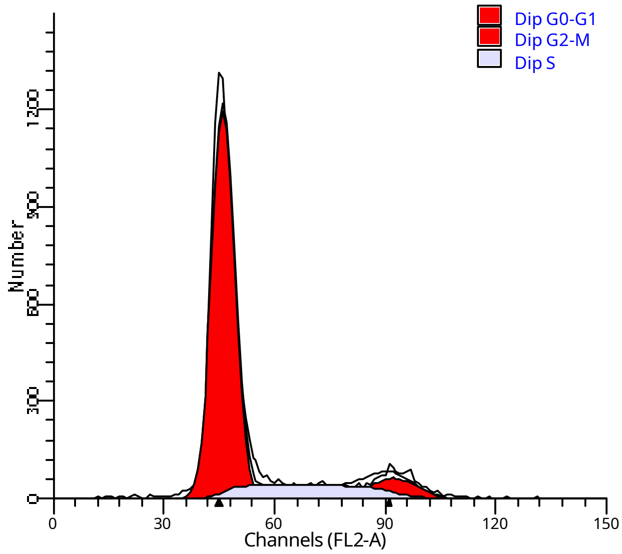

Date: 04-Jan-70 File: Data.013
Model:
%G0-G1: 76.93 at 46.02
%S: 15.02
%G2-M: 8.05 at 92.05
%CV: 6.63
RCS: 4.809

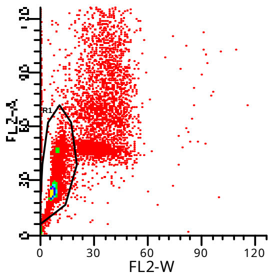

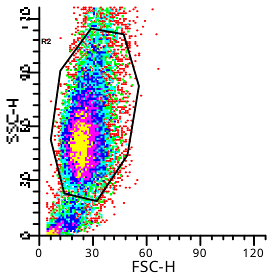

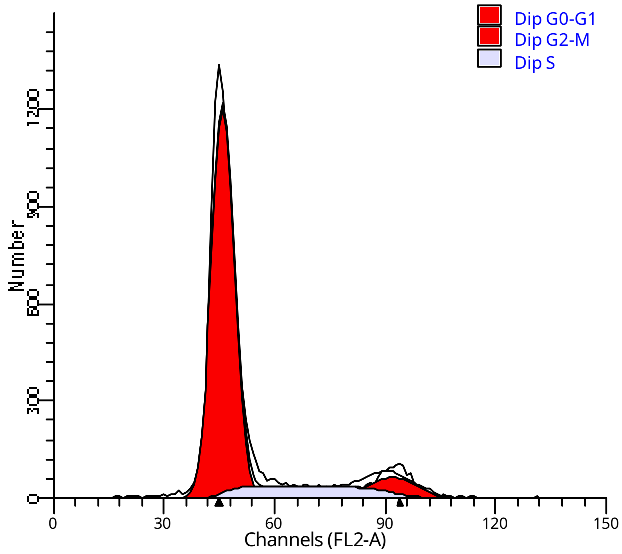

Date: 04-Jan-70 File: Data.014
Model:
%G0-G1: 78.02 at 45.93
%S: 13.57
%G2-M: 8.41 at 91.87
%CV: 6.67
RCS: 5.307

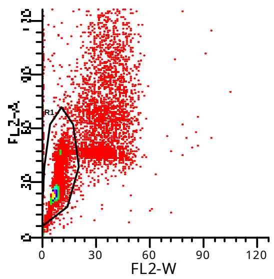

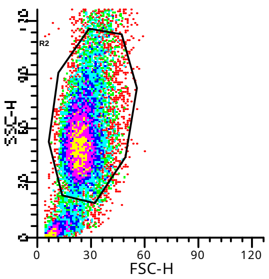

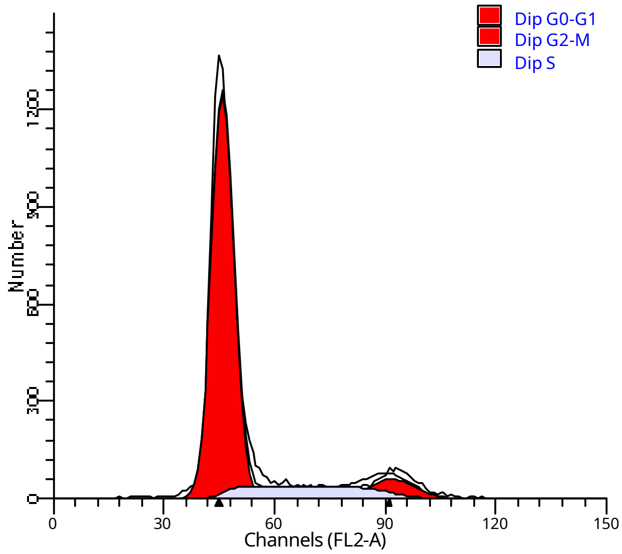

Date: 04-Jan-70 File: Data.015
Model:
%G0-G1: 78.72 at 45.90
%S: 13.81
%G2-M: 7.48 at 91.79
%CV: 6.52
RCS: 5.355

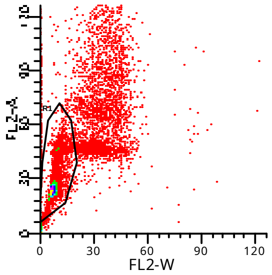

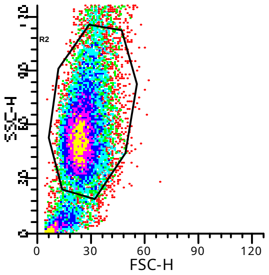

ModFitLT V3.0(PMac)

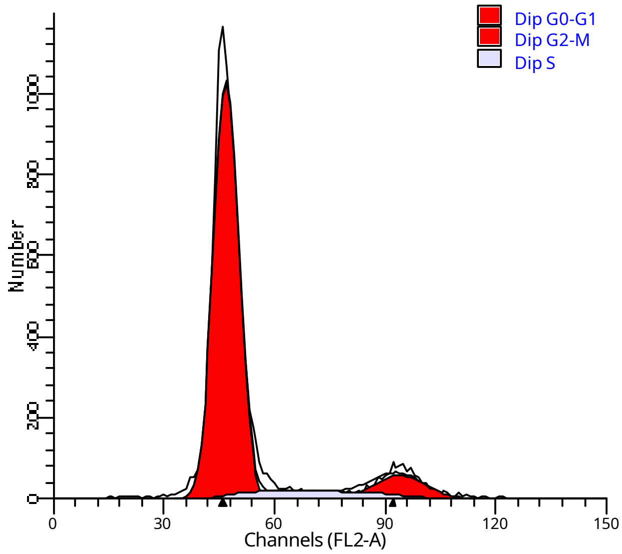

Date: 04-Jan-70 File: Data.016
Model:
%G0-G1: 83.38 at 46.83
%S: 7.48
%G2-M: 9.14 at 93.66
%CV: 7.19
RCS: 4.853

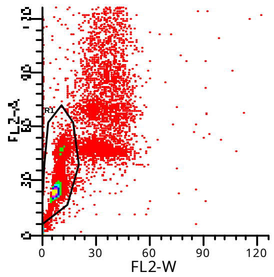

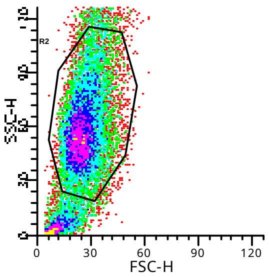

ModFitLT V3.0(PMac)

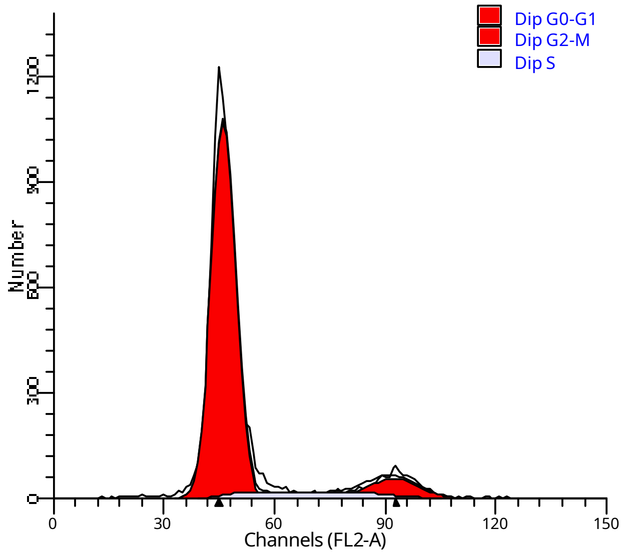

Date: 04-Jan-70 File: Data.017
Model:
%G0-G1: 83.26 at 46.11
%S: 7.86
%G2-M: 8.88 at 92.23
%CV: 7.10
RCS: 4.424

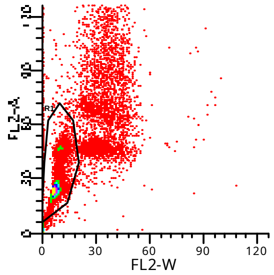

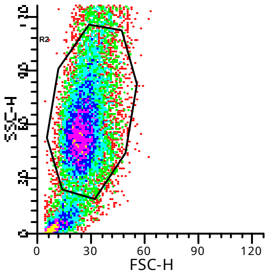

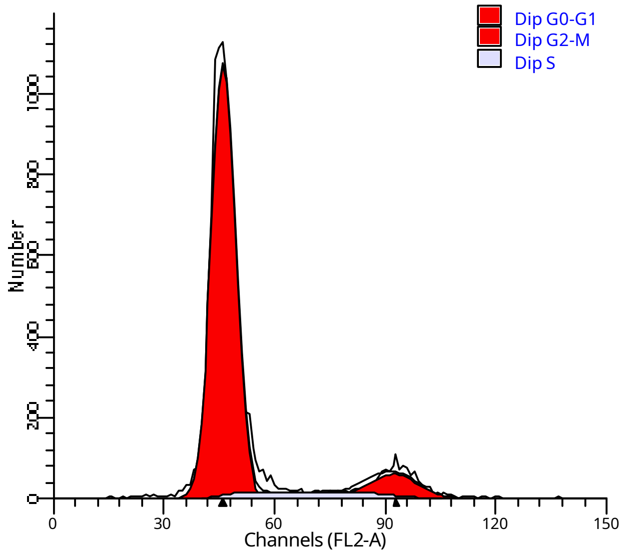

Date: 04-Jan-70 File: Data.018
Model:
%G0-G1: 83.58 at 46.11
%S: 7.04
%G2-M: 9.38 at 92.21
%CV: 7.04
RCS: 4.822

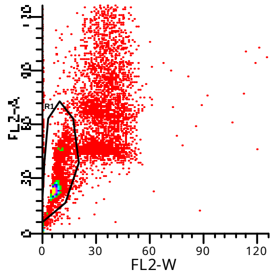

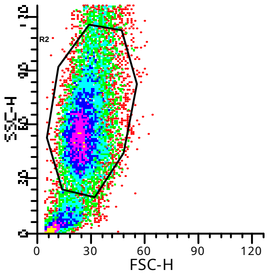

ModFitLT V3.0(PMac)

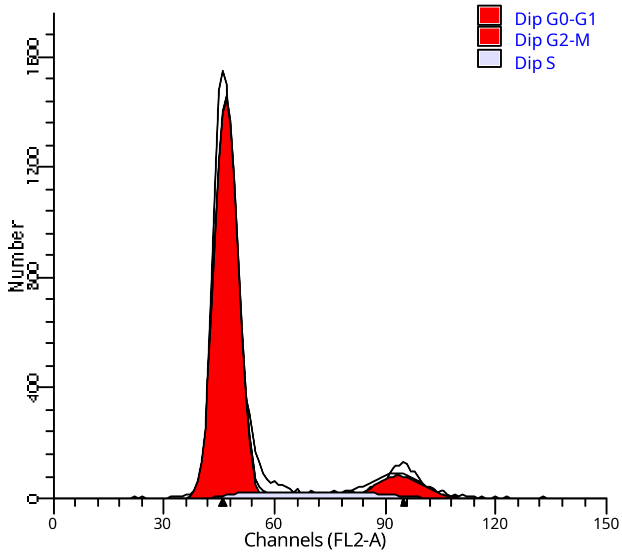

Date: 04-Jan-70 File: Data.019
Model:
%G0-G1: 83.36 at 46.85
%S: 7.22
%G2-M: 9.42 at 93.70
%CV: 6.64
RCS: 7.038

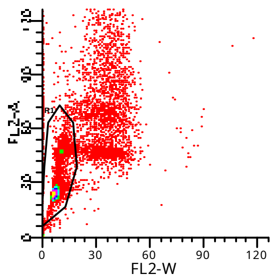

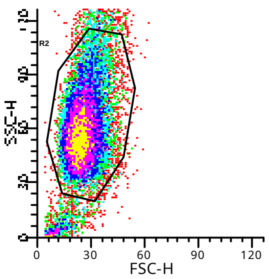

ModFitLT V3.0(PMac)

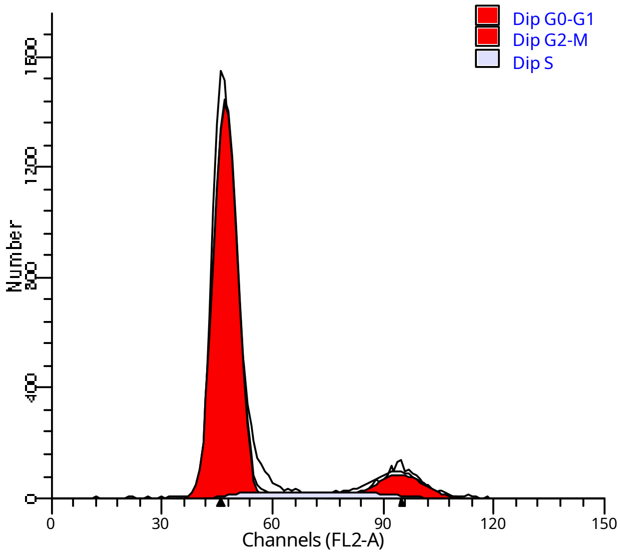

Date: 04-Jan-70 File: Data.020
Model:
%G0-G1: 82.69 at 47.21
%S: 7.24
%G2-M: 10.07 at 94.42
%CV: 6.63
RCS: 6.929

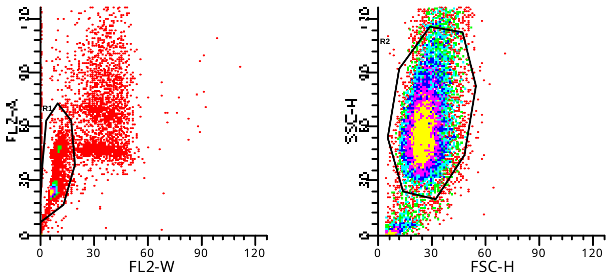

ModFitLT V3.0(PMac)

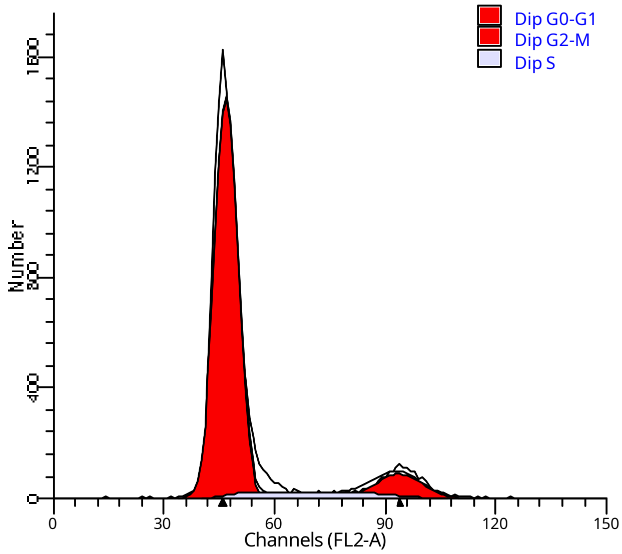

Date: 04-Jan-70 File: Data.021
Model:
%G0-G1: 82.80 at 46.84
%S: 7.14
%G2-M: 10.06 at 93.68
%CV: 6.69
RCS: 6.412

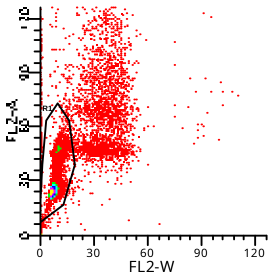

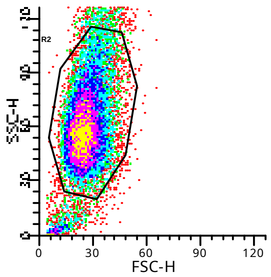

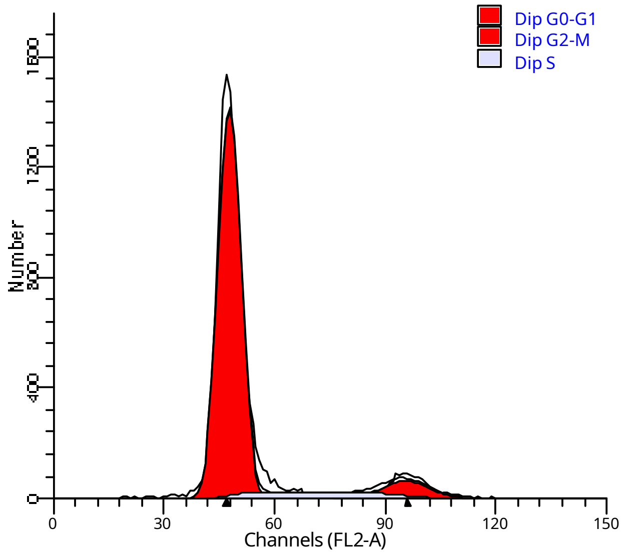

Date: 04-Jan-70 File: Data.022
Model:
%G0-G1: 84.71 at 47.76
%S: 7.66
%G2-M: 7.62 at 95.52
%CV: 6.38
RCS: 5.673

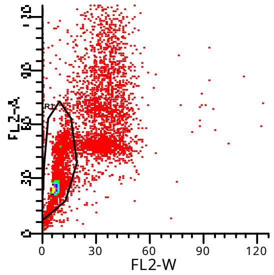

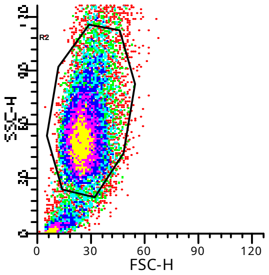

ModFitLT V3.0(PMac)

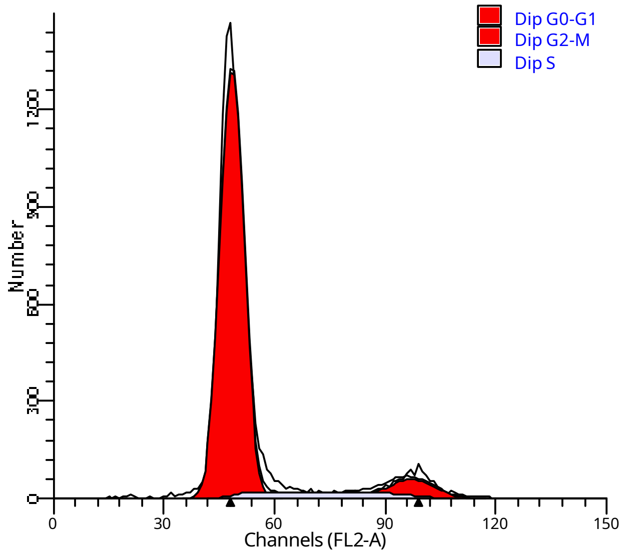

Date: 04-Jan-70 File: Data.023
Model:
%G0-G1: 84.91 at 48.44
%S: 7.68
%G2-M: 7.41 at 96.88
%CV: 6.53
RCS: 5.839

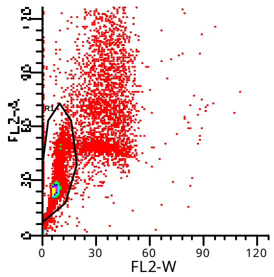

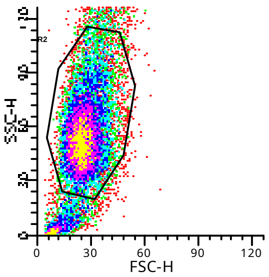

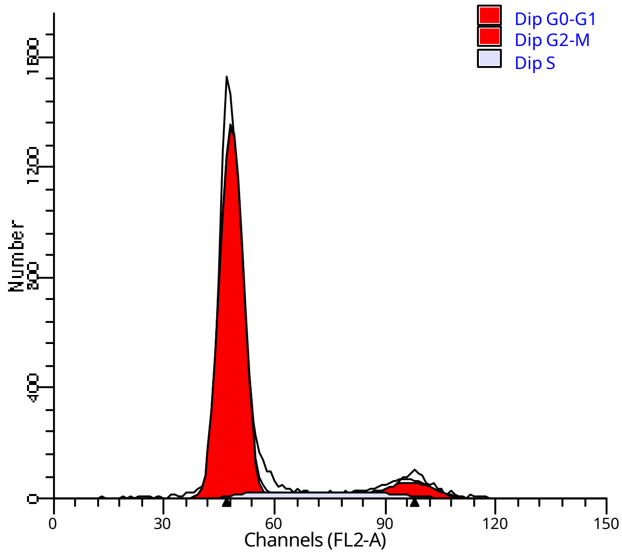

Date: 04-Jan-70 File: Data.024
Model:
%G0-G1: 84.33 at 48.27
%S: 8.25
%G2-M: 7.42 at 96.53
%CV: 6.37
RCS: 6.763

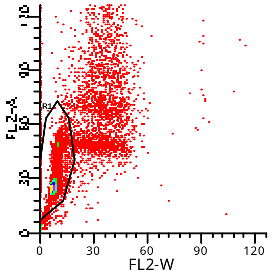

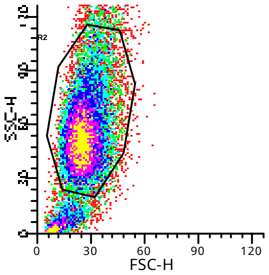

Supplement: Supplementary file 15 [file elife-85246-fig4-data3.docx]
